# Discovery of Nylon 11 ingestion by mealworm (*Tenebrio molitor*) larvae and detection of monomer-degrading bacteria in gut microbiota

**DOI:** 10.3934/microbiol.2022039

**Published:** 2022-12-26

**Authors:** Amelia Leicht, Jocelyn Gatz-Schrupp, Hisako Masuda

**Affiliations:** School of Sciences, Indiana University Kokomo, 2300 S Washington St., Kokomo, IN, USA

**Keywords:** Nylon 11, plastics biodegradation, gut microbiome, *Tenebrio molitor*, mealworm

## Abstract

Nylon 11, which can be found in many commercial products, is a synthetic plastic that has previously been considered non-biodegradable. Increasing nylon 11 and other plastics in landfills and in the environment pose an environmental concern. Recent studies on plastic biodegradation revealed that initial mechanical fragmentations increase the rate of degradation. In this study, we discovered that the larvae of mealworm (*Tenebrio molitor*) can masticate nylon 11 film at the rate of 0.25 ± 0.07 mg per fifty larvae per day. The body mass of larvae did not differ from that of starvation control while feeding on nylon 11. Comparison of gut microbiota in nylon-fed and starving larvae showed a shift in composition. There was a significant variation in community composition among the nylon 11-fed experimental groups, suggesting that many organisms are capable of metabolizing nylon 11 fragments and/or possess a growth advantage in a nylon-fed gut environment. We also discovered that a significant fraction of gut microbiome of control larvae is capable of metabolizing nylon 11 monomer (11-aminoundecanoic acid) even in the absence of prior exposure to nylon 11. This is the first study demonstrating ingestion of nylon polymers by invertebrates, and our results suggest the potential of mealworm larvae for nylon 11 biodegradation applications.

## 1. Introduction

Nylon 11 is a bio-based synthetic plastic widely used in many commercial applications, such as tubing or coatings for oil pipelines, electric cable sheaths, sporting goods and catheters [1]. It is synthesized by the polymerization of 11-aminoundecanoic acid (11-AUA) [2]. Since 11-AUA is derived from a biomass source (i.e., castor oil) rather than from fossil-fuels, it is classified as a bio-based plastic [2]. However, it has been considered non-biodegradable [3]. This is due to extensive hydrogen bonding between nylon 11 polymers, which give rise to its excellent mechanical and thermal properties [4]. While nylon 11 depolymerization can occur abiotically at high temperatures with acid treatment [5], biological degradation at ambient conditions has not been achieved. Despite their extensive commercial use, the current lack of recycling and degradation methods destine their waste to landfills. Development of cost effective, safe remediation methods are desired.

Recent studies demonstrated that the biodegradation rates of crystalline plastics can be accelerated by first introducing chemical and physical alterations (e.g., size, form, surface area and hydrophobicity) [6]–[8]. Among various biotic and abiotic mechanisms, the use of invertebrates has attracted attention as a promising method in light of the green chemistry principle [9]. Multiple invertebrates have been found to ingest biological and non-biological polymers, leading to fragmentation and deterioration [9]–[11]. In some organisms, ingested plastics increase their body mass and/or survival rate [12]–[15]. In other cases, ingested plastics do not alter these factors [11],[16],[17]. Negative effects of plastic ingestion on organisms have also been detected [10]. The presence of plastic metabolizing bacteria in gut microbial communities and their importance in plastic metabolism has been demonstrated [16],[18],[19].

Nylons are polyamides with various chain lengths [1]. The biodegradation of nylons has been examined mainly using nylon 6 and nylon 6,6 [20],[21]. Oligomers of nylon 6 and nylon 6,6 are degraded by various bacterial strains, and enzymes responsible for degradation have been characterized [22],[23]. While the degradation of nylon oligomers has been extensively studied, the biodegradation of nylon in polymer form by organisms has not been described. Additionally, to the best of our knowledge, mastication of large nylon polymers by invertebrates has not been reported.

The study of nylon 11 biodegradation is scarce. Two earlier biodegradation studies were performed with nylon 11/chitosan mixture or nylon 11/L-alanine co-polymers but not with pure nylon 11 [24],[25]. The nylon 11/chitosan mixture lost mass when buried in soil, suggesting that nylon 11 can be bioprocessed [25]. However, no organism responsible for mastication or degradation was identified.

Our previous study of nylon 11 enrichment culture with soil microorganisms led to the identification of 11-aminoundecanoic acid-degrading bacteria [26], suggesting that one of the intermediates of nylon 11 degradation is 11-AUA. In this study, the ability of mealworm larvae to fragment nylon 11 polymers was tested and confirmed. Nylon 11 did not exert a negative impact on larvae's survival rate. The presence of 11-AUA metabolizing bacteria among their gut microbiome suggests a possibility that their gut microbes can further process nylon fragments. This is the first study demonstrating nylon polymer ingestion by invertebrates with potential biodegradation implications.

## 2. Materials and methods

### 2.1. Materials and organisms

Nylon 11 and other chemicals were purchased from Sigma-Aldrich (Merck KGaA, Darmstadt, Germany). Deionized water was used for creating growth media. DEPC-treated water was used for molecular experiments. Bacterial strains were grown on defined media [27] supplemented with 11-aminoundecanoic acid as a sole source of carbon and nitrogen [26]. Bacteria were also grown on modified tryptic soy media (TSA) consisting of 4.25 g·L^−1^ tryptone, 0.75 g·L^−1^ soytone, 0.625 g·L^−1^ dextrose, 1.25 g·L^−1^ sodium chloride and 0.625 g·L^−1^ dipotassium phosphate. For solid media, 15 g·L^−1^ agar was added. Nylon 11 was heated then stretched to approximately 0.1 mm thickness [4]. Larvae of *Tenebrio molitor* were purchased from Ward's Science (Rochester, NY, USA).

### 2.2. Incubation of Nylon 11 with mealworm (Tenebrio molitor) larvae

Larvae from the single initial supply were separated into six sets of 50 individuals. On day 1, 5 grams of nylon 11 was added to three of the sets. No additional food or water source was added. The other three larvae sets were left under starvation (no food or water) and served as a negative control. Another set of three boxes, each containing 100 larvae, were also incubated with nylon 11. Larvae were incubated at room temperature. The masses of nylon 11 and the larvae were monitored for the span of 32 days. The microscopic images of the nylon 11 pieces were recorded using an Olympus BX60 microscope coupled with DP70 (Olympus, Tokyo, Japan), or by Olympus CKX41 and recorded via Mic-Fi digital microscope (Italeco S.R.L., Italy).

### 2.3. Growth substrate analysis of gut bacteria

50 mg of fecal matter collected at the end of the study was resuspended in 1 mL 50 mM phosphate buffer (pH 8). The solutions were diluted to 10^−1^, 10^−2^, 10^−3^, 10^−4^ and 10^−5^ concentrations and spot plated as previously described [28]. Solutions were spotted on both TSA and defined media supplemented with 11-AUA as a sole source of carbon and nitrogen [26]. Cells were incubated at 30 °C.

### 2.4. Illumina sequencing of 16S rRNA sequencing of microbiota in mealworm larvae feces

The DNA from the mealworm feces was extracted using E.Z.N.A. stool DNA kit (Omega-Biotek Inc., GA, USA) according to the instructions. RNA was removed by incubation with RNase at 37 °C. The quality and the quantity of total RNA was confirmed by gel electrophoresis and absorbance at 260 and 280 nm. The sequences of the V3-V4 region of the 16S rRNA gene were obtained by Illumina MiSeq sequencing. Sequencing was performed by Psomagen Inc. (MD, USA).

### 2.5. Bioinformatic and statistical analyses

The Illumina reads were assembled and processed using *micca* [29]. Barcode and primer sequences, as well as low quality reads with average quality score of less than 25, were removed. Assembled reads with 97% sequence identity and higher were classified into operational taxonomic units (OTU). Ribosomal Database Project (RDP) classifier [30] was used to assign taxonomic information to each OTU. Reads were further analyzed by the following R packages: *phyloseq, vegan, biostrings, reshape, ape, scales, picante, ggpubr* and *gridExtra* [31]–[39]. The principal coordinate analysis (PCoA) method was employed to analyze the dissimilarity. PcoA, heatmap and bar plots were constructed using *vegan*, *ggplot2* and *ggbiplot* in R [40],[41]. The t-test was employed to detect statistically significant differences between two groups.

## 3. Results and discussion

### 3.1. Ingestion of nylon 11 by mealworm larvae

**Figure 1.**
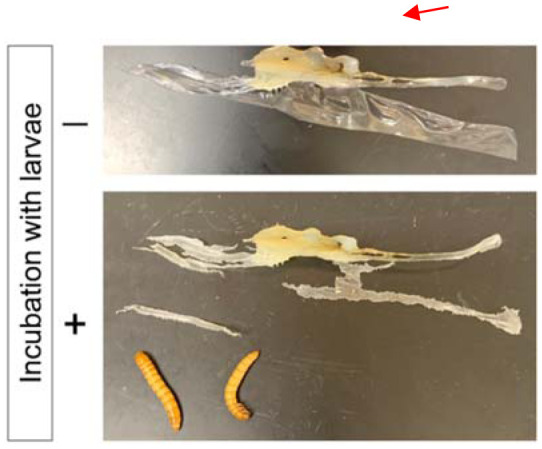
Mastication of stretched Nylon 11 film. Nylon films with and without incubation with fifty mealworm larvae are shown. Representative larvae were photographed for size comparison. The red arrow indicates the area where mastication was not observed.

**Figure 2.**
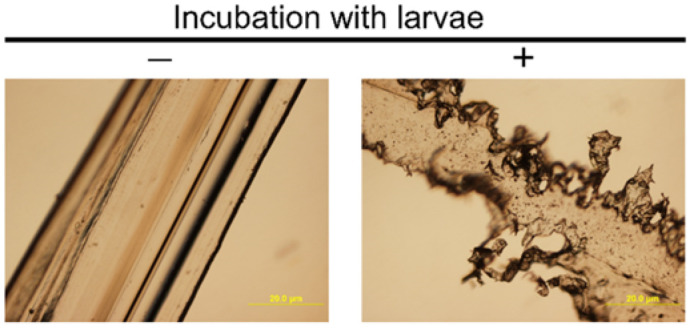
Microscopic observation of intact nylon 11 filament. Nylon filaments with and without incubation with larvae are shown.

Since larvae did not ingest intact nylon 11 pellets (3 mm in diameter), nylon 11 was melted and stretched to a thin film. Ingestion by larvae at a measurable rate was observed in the areas of reduced thickness (Figure 1). The thickness seems crucial for ingestion, as the regions of stretched nylon 11 that remained thick (i.e., > 1 mm) were not ingested by the larvae even after one month of incubation (Figure 1 - red arrow). The thickness of the film for which ingestion was attainable was estimated via visual observation by light microscope. As shown in Figure 2, film of less than 50 µm thickness allowed for the rapid formation of numerous indentations by the larvae in less than 2 days.

**Figure 3.**
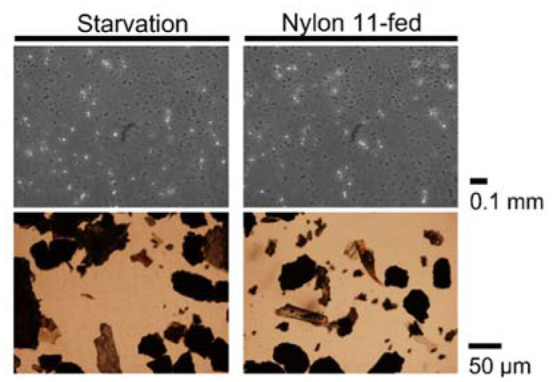
Fecal matter of larvae with and without nylon 11 feeding.

To examine the fate of ingested nylon 11, we first observed fecal matter under the microscope. Microscopic investigation did not identify fragments distinctive from what was found in the starvation control (Figure 3). Additionally, we analyzed the chemical compositions of feces by thin-layer chromatography, gel permeation chromatography and high-pressure liquid chromatography. Expected peaks corresponding to nylon 11 polymer, oligomer or monomer were not identified (data not shown).

**Figure 4.**
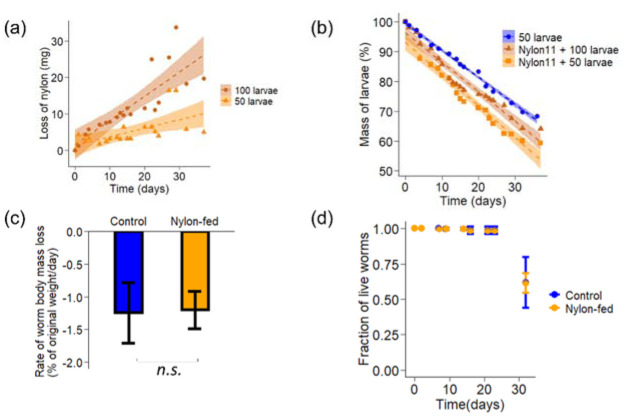
Changes in mass of nylon 11 and effects on larvae's mass and their survival rate. (a) Mass of lost nylon 11 while incubated with fifty (in orange) or one hundred (in red) larvae and (b) the % of initial mass of larvae over study period. Control larvae incubated without nylon 11 are shown in blue. c) Rates of larvae mass change and d) survival curve.

Despite the fact that no other food or water was added, a vast majority of larvae lived and continued to ingest nylon 11 for the 32-day study period (Figure 4a). The rate of nylon ingestion was calculated from the slope of the graphs. The rate of nylon mass decrease depended on the number of worms incubated. The rates of nylon mass decrease were 0.25 mg·day^−1^ and 0.66 mg·day^−1^ for incubation with 50 and 100 worms, respectively. The rate nearly doubled when the number of worms was increased two-fold. This suggests that increasing the number of worms per mass of nylon 11 and/or increasing the accessible edges could further increase the rate of fragmentation.

### 3.2. Effects of nylon 11 on body mass change and survival curve

To study whether ingested nylon 11 supports larvae growth and/or exerts negative impacts, the change in body mass and survival rate of larvae were analyzed. The body weight declined continuously during the study period (Figure 4b). The rate of body mass loss was indistinguishable between nylon-fed and starvation control: 1.20 ± 0.28% and 1.24 ± 0.46% of initial mass per day, respectively (Figure 4b,c). The body mass loss was solely due to the loss of body mass in individual organisms, as the number of live individuals remained constant. The survival curves were also indistinguishable between worms with and without the nylon 11 addition (Figure 4d). After approximately 32 days, some larvae started metamorphosis, and others died. Since nylon 11 ingestion does not impact the survival rate nor body mass, nylon 11 seems to neither provide sufficient energy nor exert toxicity on larvae. Further studies to increase the efficiency of nylon ingestion would allow us to ascertain whether larvae or gut bacteria metabolize nylon 11 fragments.

### 3.3. Gut microbial composition change in nylon 11-fed larvae

**Figure 5.**
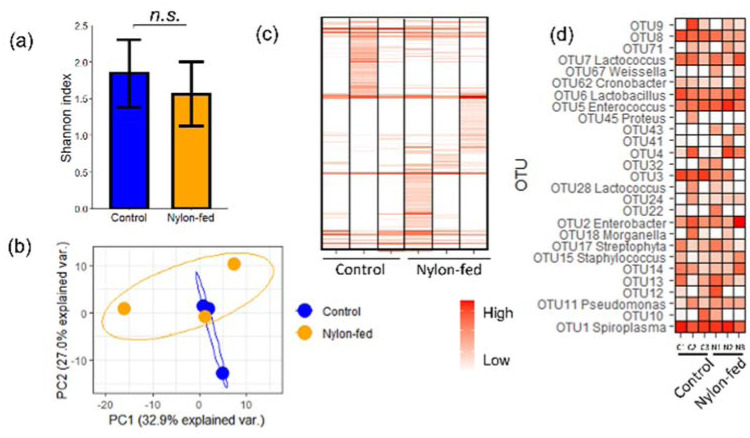
Gut microbiome of larvae studied by Illumina sequencing of the V3-V4 region of the 16S small subunit rRNA gene sequences. The 16S small subunit rRNA gene sequences were clustered into OTUs, and alpha and beta diversities were measured as (a) Shannon diversity index and (b) principal component analysis of unweighted Unifrac distance, respectively. *n.s*. indicates a lack of difference with statistical significance (t-test; *p* value < 0.05). A heatmap of (c) entire OTU table and (d) 27 most abundant OTUs with taxonomic information.

To study whether fragmented nylon 11 impacted gut bacterial composition, we investigated the gut microbiota via high-throughput sequencing of 16S rRNA gene fragments. Sequences with 97% identity were grouped into OTUs. A total of 266 OTUs were identified, and taxonomic information was assigned. As shown in Figure 5a, the alpha diversity, measured by Shannon index, did not differ between control and nylon-fed samples.

The PCoA plot revealed the separation of control and nylon-fed bacterial compositions (Figure 5b). To better understand the dissimilarity, a relative abundance of all OTU (Figure 5c) and top 27 abundant sequences (Figure 5d) were compared. There are sequences commonly found in all control and nylon-fed worms' feces, including OTU1 (*Spiroplasma*), OTU2 (*Enterobacter*), OTU5 (*Enterococcus*), OTU6 (*Lactobacillus*), OTU7 (*Lactococcus*) and OTU8 (*Enterobacteriaceae*). One of the control samples (C2) and two nylon-fed samples (N1 and N3) contained many unique OTUs with low to medium abundance (Figure 5c).

There were sequences that were more abundant in controls (OTU3 and OTU8) than in nylon-fed groups with weak statistical significance (*p* < 0.1). We did not find any sequences that were significantly more abundant in all nylon-fed samples than in control with *p* < 0.05. However, we identified sequences that were uniquely higher in one of the nylon-fed sample than controls. OTU22 (Firmicutes, Bacillales) composed 2.3% of the N1 sequences while it was 0.02% of controls (400-fold enrichment). OTU13 (unknown), OTU17 (*Streptophyta*), OTU32 (unknown) and OTU12 (Clostridiaceae) were also uniquely enriched in the N1 sample (3.0, 2.2, 3.3 and 122-fold, respectively). OTU5 (Enterococcus) was enriched 2.6-fold in the N2 sample. In the N3 sample, OTU15 (*Staphylococcus*), OTU2 (*Enterobacter*) and OTU14 (Enterobacteriaceae) were enriched by 6.3, 5.2 and 2.6-fold, respectively.

Distinctive OTUs were enriched in each fecal sample from nylon-fed worms (Figure 5c), despite the fact that worms from the same shipment were used. The reason for why different groups of bacteria were enriched in replicate samples remains unknown. Also, the composition of one of the nylon-fed samples (N2) was nearly identical to two control samples (C1 and C3). The reasons for the shift in microbiota composition, as well as variability of composition between the nylon-fed groups, will be investigated in future studies. Interestingly, many of the abundant/enriched sequences did not have a high similarity to known bacterial sequences in the public database at the level of genus, family or in some cases higher level of phylogenetic classifications (e.g., OTU2, OTU13, etc.). Their contribution to nylon metabolism is of interest, as some sequences belong to a phylogenetic group with known strains with plastic-degrading ability (e.g., Firmicutes, Proteobacteria and Actinobacteria) [18],[42]–[44].

### 3.4. A lack of enrichment of 11-AUA degrading bacteria in feces

**Figure 6.**
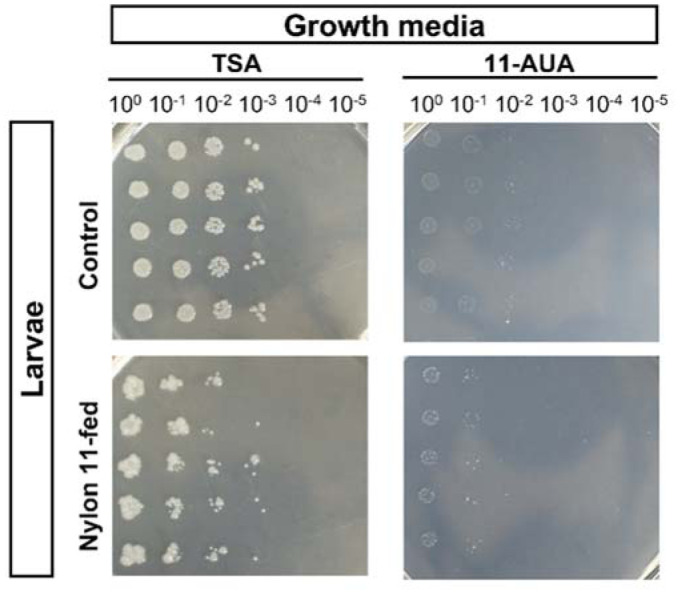
Growth of gut bacteria on rich and defined media with 11-AUA. Fecal matter from control and nylon 11-fed larvae were diluted to designated factors and spot plated on TSA and defined media supplemented with 11-AUA as the sole source of carbon and nitrogen.

**Figure 7.**
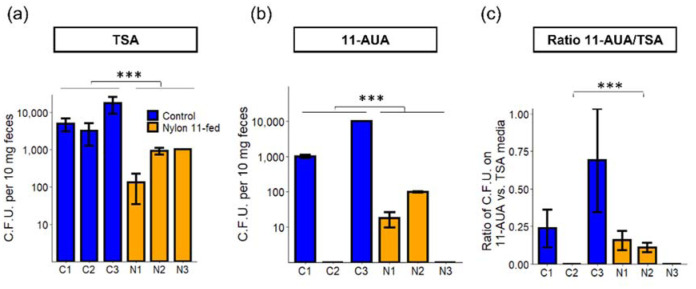
Comparison of colony forming units on rich media vs. defined media with 11-AUA. C.F.U. per 10 mg of feces were measured on (a) TSA and (b) defined media with 11-AUA. (c) The ratios of C.F.U. on TSA and 11-AUA media were compared. The statistical analysis was performed by t-test. *** indicates statistical significance (*p* value < 0.05).

To further examine the impact of nylon-ingestion on the gut microbiome of the larvae, the number of total culturable bacteria on selective and rich media was analyzed (Figures 6,7). On rich media (TSA), the colony forming unit (C.F.U.) was slightly higher for control than nylon-fed samples (*p* value = 0.04). Next, to see if there were any changes in community composition that were linked to nylon 11 metabolism, we analyzed the C.F.U. on minimal media containing 11-AUA as a sole source of carbon and nitrogen. We reasoned that if ingested nylon 11 were fragmented into oligomers and/or monomers and available for bacterial metabolism, then bacteria capable of utilizing nylon 11 monomer (11-AUA) would be enriched. To our surprise, the number of 11-AUA degrading bacteria was high even in control samples (Figure 7b). Also, a higher number of 11-AUA metabolizing bacteria were present in control samples than in nylon-fed samples for the same amount of feces. C.F.U. values for the C1 and C3 samples were 1,000 and 10,000, while those of the N1 and N2 samples were 18 and 98, respectively. To rule out the possibility that the higher C.F.U. on 11-AUA media was not simply due to the higher amount of total culturable bacteria, the ratio of C.F.U. on 11-AUA media vs. TSA was calculated (Figure 7c). The results showed that control samples contained a higher fraction of bacteria capable of degrading 11-AUA than nylon-fed samples: 23% and 69% of the C1 and C3 samples were 11-AUA metabolizing bacteria, while 16% and 11% were for the N1 and N2 samples, respectively.

A lack of increase of 11-AUA metabolizing bacteria in feces of nylon-fed mealworms could be due to the following reasons: 1) The ingested nylon 11 fragments were not available for metabolism by the gut bacteria; 2) nylon 11 was metabolized via a pathway that does not involve 11-AUA as an intermediate; or 3) resident bacteria were metabolizing 11-AUA, but their relative abundance did not change. We would like to note that this method does not allow us to detect a change in non-culturable populations. It is possible that non-culturable populations (i.e., organisms detected in metagenomic analysis) play a significant role in nylon fragment metabolism in the gut.

The presence of 11-AUA degrading bacteria in the gut of control larvae is unique, compared to our separate experiment with the adult mealworm beetle. In the adult beetles study, the starvation control did not harbor 11-AUA degrading bacteria. Ingestion of nylon 11 increased the 11-AUA degrading bacteria by 10,000-fold. Differences in the gut microbiome of larvae and adult, despite that they both ingest nylon 11, is of interest. We plan to study further to decipher the optimum strategies for nylon 11 biodegradation using mealworms at different stages of life.

## 4. Conclusions

In this study, we have shown that mealworm larvae can ingest nylon 11. 0.25 mg of nylon 11 films was ingested by fifty worms per day for a period of 32 days. Absence of visible fragments in their fecal matter suggests that nylon 11 pieces were fragmented to a very small size or completely mineralized. The shift of gut microbial community composition was observed. Interestingly, mealworms devoid of previous exposure to the nylon 11 carried a significant number of monomer-degrading bacteria. Our results illuminate a potential use of mealworm larvae for nylon 11 bioremediation applications.
